# Impact of leadership styles on patient satisfaction with nursing care quality in public hospitals: A cross-sectional study

**DOI:** 10.1097/MD.0000000000041670

**Published:** 2025-03-14

**Authors:** Amira Yahia Boshra, Fatima Ahmad Aseeri, Sharifa Alasiry, Mehrunnisha Ahmad, Aksh Chahal, Gopal Nambi, Mohammad Abu Shaphe, Mohammad Sidiq, Abhishek Sharma, Faizan Kashoo

**Affiliations:** a College of Nursing, Majmaah University, Al-Majmaah, Saudi Arabia; b Security Forces Hospital Program, Saudi Arabia; c Department of Physiotherapy, School of Allied Health Sciences, Multi-Disciplinary Research and Development Cell (G-MRDC) Galgotias University, Greater Noida, Uttar Pradesh, India; d Department of Physical Therapy and Health Rehabilitation, College of Applied Medical Sciences, Prince Sattam Bin Abdulaziz University, Al Kharj, Saudi Arabia; e Department of Physical Therapy, College of Nursing and Health Sciences, JAZAN University, Jazan, Saudi Arabia; f Arogyam Institute of Paramedical and Allied Sciences, Arogyam Medical College and Hospital (affiliated with H.N.B. Uttarakhand Medical Education University) Roorkee, District-Haridwar, Uttarakhand, India; g Department of Physical Therapy and Health Rehabilitation, College of Applied Medical Sciences, Majmaah University, Al-Majmaah, Saudi Arabia.

**Keywords:** cross-sectional study, leadership, nursing, patient satisfaction

## Abstract

This cross-sectional study investigates the relationship between nursing leadership styles and patient satisfaction in 4 public hospitals in Saudi Arabia. The nonrandom convenience sampling method was used with 150 nurses who were involved in caring for 180 patients from 4 different hospitals in the central region of Saudi Arabia. Nursing leadership styles were assessed from nurses using the Multifactor Leadership Questionnaire, while patient satisfaction was measured using the Patient Satisfaction with Nursing Care Quality Questionnaire. Passive-avoidant (PA) leadership style was most prevalent among nurse managers (62.6%, n = 94) and was associated with the lowest patient satisfaction (*m* = 1.7, SD = 0.1), with ratings ranging from 0 (low satisfaction) to 4 (high satisfaction). Analysis of variance (ANOVA) revealed a statistically significant difference in patient satisfaction scores among leadership styles (*F* (3, 176) = 25.421, *P* < .001). The transactional leadership demonstrated higher mean scores (*m* = 2.7) compared to both the PA leadership style (*m* = 2.1) (MD = 0.60, SE = 0.071, *P* < .001, 95% CI [0.42, 0.79]) and the transformational leadership leadership style (*m* = 2.2) (MD = 0.54, SE = 0.10, *P* = .004, 95% CI [0.21, 0.88]). Conversely, the PA leadership style had lower mean scores (*m* = 2.1) than both the transactional leadership group (MD = 0.60, SE = 0.07, *P* < .001, 95% CI [0.42, 0.79]) and the outcome leadership style (*m* = 2.5) (MD = 0.37, SE = 0.09, *P* = .008, 95% CI [0.09, 0.65]). In Saudi Arabian public hospitals, PA leadership style was found to be the most prevalent among nurse managers, associated with the lowest levels of patient satisfaction. These findings underscore the importance of nursing leadership styles in affecting patient satisfaction. Further research is needed to explore the specific strategies that can be employed to foster effective leadership styles for improved patient outcomes.

## 1. Introduction

The quality of nursing services and leadership styles play a pivotal role in patient satisfaction, affecting the overall success of healthcare services.^[1]^ Research reports positive associations between effective leadership styles and increased patient and staff satisfaction.^[2–4]^ Effective leadership has been identified as a critical component leading to successful healthcare outcomes.^[5]^ A recent study reported the influence of leadership styles on nurses’ job satisfaction, turnover, and patient care quality.^[6]^

Leadership styles include a range of practices for effectively guiding and motivating individuals and teams.^[7]^ Different types of leadership styles in nursing, as described by Bass and Avolio in the multifactor leadership questionnaire (MLQ),^[8]^ include transformational leadership (TF), transactional leadership (TS), outcome leadership, and passive avoidance. TF is characterized by its emphasis on inspiring and motivating teams through the presentation of a compelling vision.^[9]^ This is frequently exemplified by charismatic and visionary leaders who possess a strong passion for attaining their objectives. This particular approach cultivates innovation, individual development, and a sense of direction among members of a team, leading to elevated team morale, enhanced creativity, and improved overall performance.^[9]^ On the contrary, TS prioritizes the establishment of explicit expectations, provision of rewards, and imposition of consequences. Leaders exhibiting this style tend to possess traits such as organization, task orientation, and a strong focus on procedural aspects.^[10]^ The team’s interactions are characterized by a transactional nature, wherein they adhere to established rules and are motivated by rewards. This approach effectively upholds order within the team and ensures the successful completion of tasks. Outcome Leadership is a leadership approach that focuses on attaining precise outcomes and results through the utilization of goal-oriented and data-driven decision-making processes.^[11]^ Leaders who adopt this particular style exhibit the ability to establish unambiguous goals and objectives, thereby fostering a culture that prioritizes achieving desired outcomes and upholding responsibility. In contrast, Passive Avoidance Leadership is characterized by a tendency to refrain from actively engaging in leadership responsibilities and decision-making processes.^[12]^ Leaders exhibiting this style demonstrate passivity and indifference, providing limited guidance and direction. This approach has the potential to result in various negative outcomes, including confusion, a lack of clear objectives, and a decrease in team motivation.^[13]^ Moreover, research by Den Breejen-de Hooge et al (2021)^[14]^ reported the importance of stronger leadership among nurses to enhance healthcare standards and patient safety. The latter study reported the association between leadership and the quality of care, with nurse characteristics playing a significant role in quality improvement in clinical settings. Alloubani et al (2019)^[15]^ reported a positive correlation between the TF style and leadership outcomes, as well as nursing care quality. Zaghini et al (2020) suggest that nurses’ perceptions of care are influenced by leadership, organizational context, and nursing behaviors.^[16]^

The cultural attributes of Saudi society, including a strong emphasis on hierarchical structures, collectivism, adherence to Islamic principles, gender segregation, and a focus on family-centered care, have the potential to shape the prevailing leadership styles within the nursing profession in Saudi Arabia.^[17]^ The success of nursing leaders in Saudi Arabia will depend on their ability to modify and synchronize their leadership strategies with the prevailing cultural values, all while maintaining a commitment to providing exceptional patient care.^[18]^ To the best of our knowledge, there is a notable dearth of research articles that identify the dominant leadership styles employed by nursing managers in Saudi Arabia and their indirect influence on patient satisfaction levels. We hypothesize that leadership style will be significantly associated with patient satisfaction within Saudi Arabian public hospitals.

## 2. Materials and methods

### 2.1. Study design

Cross-sectional survey design

### 2.2. Sample size calculator

The sample size in our research study was determined by using an online calculator (https://www.calculator.net/sample-size-calculator.html). The calculation was conducted with a 95% confidence level and a margin of error of 5%, using an estimated population proportion of 50%. The sample size of 385 participants was deemed sufficient.

### 2.3. Inclusion criteria

For nurses: The study includes registered or licensed practicing nurses, and a minimum of 1 year of experience in any 1 of the following hospitals (Prince Sultan Military Medical City [PSMMC], King Fahad Medical City [KFMC], National Guard Hospital [NGH], and Prince Mohammad Bin Abdulaziz Hospital). Nurses provided informed consent and were proficient in Arabic and English language. Exclusion criteria include unwillingness to participate and part-time or temporary staff.

For patients: The patients were adults who were 18 years old and older, conscious, oriented, stable, and not severely ill or mentally disabled, and who could understand Arabic or English, from the 4 selected hospitals. Patients with the ability to provide informed consent and actively participate in the survey process are included. Patients who have stayed in the hospital for at least 24 hours are included to ensure adequate exposure to nursing care. Patients who willingly consent to participate in the study indicate their willingness to provide feedback on their experiences with nursing care.

### 2.4. Setting and study approval

This study received ethical approval from Majmaah University’s ethics committee and the Ministry of Health (MOH) with approval number IRB H-01-*R*-012, dated 5th September 2022. The study commenced its participant recruitment in October 2022, with data collection successfully concluded by June 2023. This study was conducted at prominent public hospitals situated within the Riyadh Region, encompassing the MOH hospitals. The comprehensive sample for this research was sourced from distinguished hospitals, including PSMMC, KFMC, NGH, and Prince Mohammad Bin Abdulaziz Hospital. Additionally, permission to employ the copyrighted instruments namely the MLQ was obtained from the developers. This selection of hospitals provided a diverse and representative setting for the study, offering a broad spectrum of healthcare experiences within the central region of the country.

### 2.5. Data collection procedures

**Figure 1.**
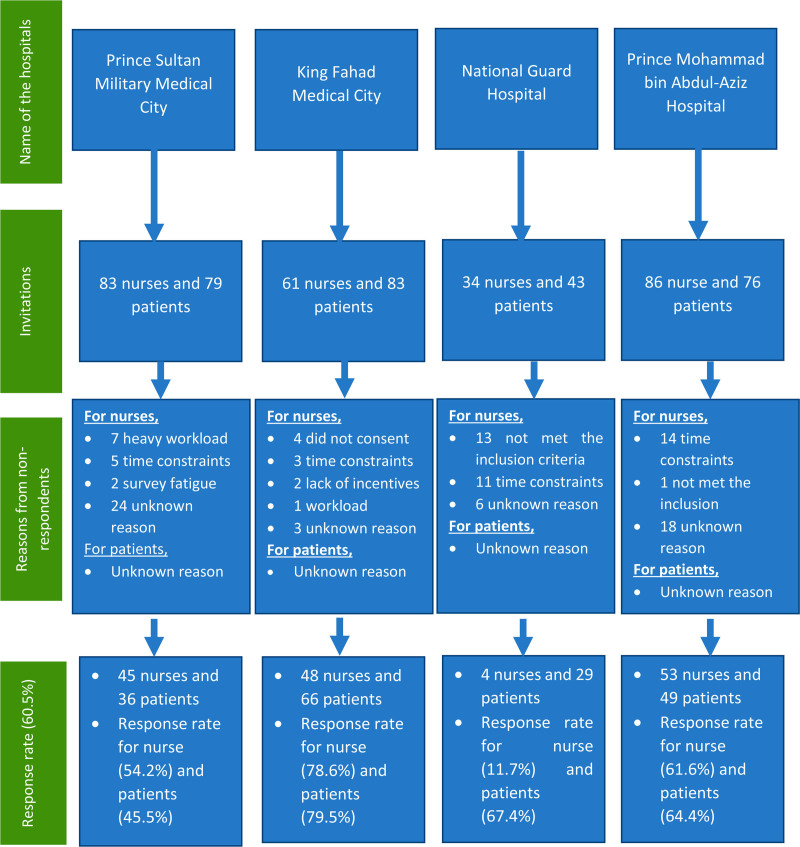
Flow chart of participant’s recruitment, nonrespondents and response rate.

Data collection procedures involved the distribution of questionnaires through online links, which were emailed and shared within relevant social media groups, with participation sought from staff nurses and patients. Additionally, with the requisite approvals from hospital administrations, the primary researcher conducted in-person visits to hospitals. During these visits, the researcher explained the study’s objectives and significance to prospective participants and invited their involvement. Each questionnaire distributed during these visits was uniquely barcoded, accompanied by a cover letter containing comprehensive information about the research study, including its title, aims, objectives, and contact details for the primary researcher. This combined approach ensured the comprehensive inclusion of participants and rigorous data collection while adhering to ethical considerations (Fig. 1).

### 2.6. Data collection tools

Data collection instruments for this study included 2 distinct online questionnaires tailored to the respective participant groups. For nurses, the MLQ was utilized. The MLQ Rater consists of 45 items: 36 questions related to leadership styles and effectiveness behaviors, while 9 questions evaluate leadership outcomes. The 12 subscales examined include idealized influence attributes, idealized influence behaviors, inspirational motivation, intellectual stimulation, individual consideration, Contingent reward (CR), management by exception (active), management by exception (passive), Laissez-Faire (LF), extra effort, effectiveness, and satisfaction.^[19]^ Responses are provided on a 5-point Likert scale, ranging from 0 (indicating that the behavior did not occur) to 4 (indicating that the leadership behavior occurred frequently, if not always). The MLQ Rater Form enables nursing staff to assess their immediate managers, while the MLQ Leader Form allows nurse managers to evaluate their leadership. The MLQ was employed to categorize leadership styles as transformational, transactional, passive-avoidant (PA), and outcome leader.^[20]^ The MLQ has been appropriately translated into the Arabic language.^[21]^ A study conducted among 600 nurses reported Cronbach α coefficients ranging from 0.78 to 0.94.^[22]^

For patients, the patient satisfaction with nursing care quality questionnaire (PSNCQQ) developed by Laschinger et al (2005) was employed.^[23]^ This tool was designed to assess the level of patient satisfaction with the quality of nursing care services. The PSNCQQ contains 22 items to assess patient satisfaction with the quality of care during their hospital stay, overall nursing care quality, and their intention to recommend the hospital to others. Respondents used a 5-point Likert scale, ranging from 0 (indicating poor) to 4 (indicating excellent), for each question in the PSNCQQ. The questionnaire was reported to be reliable and valid.^[24]^ In a study involving 226 patients, the Cronbach alpha coefficient exceeded 0.7, affirming the acceptable reliability of the research instrument.^[25]^

### 2.7. Statistical analysis

The dataset was initially analyzed for missing data (multiple imputation method) and transformations (variables combined through compute variables) to compare multiple constructs (leadership styles) assessed through the questionnaire. The study variables were subjected to descriptive statistics, which involved the computation of means, standard deviations, and frequency distributions. Chi-square was used to compare leadership styles between hospitals. Analysis of variance (ANOVA) was employed to compare scores related to patients’ satisfaction across different leadership styles. Assumption testing revealed a violation of the homogeneity of variance assumption through Levene test (Levene statistic = 2.890, *P *= .037). Given this violation, the Games-Howell procedure was employed for multiple comparisons to appropriately address the unequal variances among the groups. A sensitivity analysis was conducted to examine the relationship between leadership styles and scores on patient satisfaction, incorporating demographic data of patients as a covariate (File 1, Supplemental Digital Content, http://links.lww.com/MD/O520). The analyses were performed utilizing SPSS version 20 (Chicago) and MS Excel version 2007. A significance level of *P* < .05 was utilized to ascertain statistical significance. We assessed the underlying assumptions of the statistical tests and addressed any violations as required.

## 3. Result

### 3.1. Participants characteristics

One hundred fifty nurses were involved in caring for 180 patients who participated in this study from 4 different hospitals (Prince Mohammad Bin Abdulaziz Hospital [PMAH], NGH, KFMC, and PSMMC). Age (years) shows no statistically significant variation among the hospitals (*P* = .354). However, gender distribution reveals significant differences, with NGH and KFMC having more males and females, respectively (*P* < .05). Health status before hospital admission does not significantly differ, except for NGH, which has a higher proportion of patients in very poor health (*P*-value = .278). The health status after discharge reveals significant variations, with a higher proportion of patients at PSMMC in very poor health (*P* < .05). Furthermore, the willingness to recommend the hospital to friends/family differs significantly among the hospitals (*P* < .05) with the highest proportion of respondents at PMAH strongly disagreeing. Quality of service and nursing care were significantly rated, with NGH and KFMC performing better in both aspects (*P* < .05). Analysis of the Dominant Leadership Style indicates that PA was prevalent in PMAH and KFMC, while the TF leadership style was dominant in NGH. The nurse-care quality score also varied significantly across hospitals (*P* < .05), with NGH having the highest score, reflecting better care quality. These findings underscore the need for tailored hospital management and improvements in patient satisfaction and care quality, particularly in the context of leadership styles and nursing care. (Table 1)

**Table 1:** Demographic data of patients (n = 180).

	PMAH (n = 49)	NGH (n = 29)	KFMC (n = 66)	PSMMC (n = 36)	*P*-value
	A	B	C	D	
Age (yr)	38.1 (13.0)	36.1 (11.1)	32.1 (11.1)	34.6 (13.8)	.354
Gender					
Male	25	9^ACD^	26	15	.001
Female	24	20	40^ABD^	21	.001
Marital status					
Single	19 (38.8)	13 (44.8)	42 (63.6)	22 (61.1)	.065
Married	27 (55.1)	14 (48.3)	22 (33.3)	10 (27.8)	
Divorced/separated	3 (6.1)	2 (6.9)	2 (3.0)	4 (11.1)	
Health before admitted to the hospital					
Unsure	1 (2.0)	0	0	0	.278
Very poor	16 (32.7)	15 (51.7	35 (53.0)	16 (44.4)	
Poor	0	0	0	0	
Fair	32 (65.3)	14 (48.3)	31 (47.0)	20 (55.6)	
Good	0	0	0	0	
Excellent	0	0	0	0	
Health after discharge from the hospital					
Unsure	23 (46.9)^BCD^	1 (3.4)	1 (1.5)	1 (2.8)	.001
Very poor	19 (38.8)	15 (51.7)	36 (54.5)^ABD^	13 (36.1)	
Poor	0	0	26 (39.4)	12 (33.3)	
Fair	7 (14.3)	13 (44.8)^AC^	3 (4.5)	10 (27.8)^AC^	
Good	0	0	0	0	
Excellent	0	0	0	0	
Recommend the hospital to friends/family					
Strongly disagree	29 (59.2)	3 (10.3)	1 (1.5%)	33 (91.7)	.001
Somewhat disagree	16 (32.7)	0	33 (50.0)	3 (8.3)	
Agree	4 (8.2)	12 (41.4)	29 (43.9)	0	
Somewhat agree	0	14 (48.3)	3 (4.5)	0	
Strongly agree	0	0			
Quality of service provided by the hospital					
Poor	12 (24.5)	0	0	0	.001
Fair	24 (49.0)	0	29 (43.9)	23 (63.9)	
Good	13 (26.5)	19 (65.5)	28 (42.4)	13 (36.1)	
Very good	0	10 (34.5)	9 (13.6)	0	
Excellent	0	0	0	0	
Quality of nursing care					
Poor	8 (16.3)	0	1 (1.5)	0	.001
Fair	23 (46.9)	1 (3.4)	28 (42.4)	18 (50)	
Good	18 (36.7)	17 (58.6)	27 (40.9)	18 (50)	
Very good	0	11 (37.9)	10 (15.2)	0	
Excellent	0	0	0	0	
NCQ	1.7 (0.2)	3.1 (0.1)	2.5 (0.3)	2.4 (0.3)	.001
Dominant leadership style	PA	TS	PA	PA	

Values are presented as mean (standard deviation) or frequency (percentage); statistically significant at *P* < .05; superscripts (A, B, C, D) indicate statistically significant differences between groups based on post hoc analysis, where A denotes a significant difference from PMAH, B from NGH, C from KFMC, and D from PSMMC.

PMAH = Prince Mohammed Bin Abdulaziz Hospital, NGH = National Guard Health Affairs, KFMC = King Fahad Medical City, PSMMC = Prince Sultan Military Medical City, NCQ = nursing care quality, PA = passive-avoidant, TS = transactional leadership.

The table 2 reveals that the dominant form of leadership style among nurse managers as perceived by working nurses was PA n = 94 (62.65). The KFMC prominently featuring both Transactional (43.7%) and PA (56.2%) leadership styles, significantly distinguishing it from the other hospitals (*P* < .05). In contrast, NGH exhibits a unique leadership style characterized by absence of both Transformational and Outcome leadership styles. These divergent leadership profiles correlate with marked distinctions in patient satisfaction scores (*P* < .001), with NGH achieving the highest rating (*m* = 3.1, SD = 0.1) and PMAH the lowest (*m* = 1.7, SD = 1.0). Our findings underscore the critical role of leadership styles in shaping patient satisfaction levels, providing valuable insights for enhancing healthcare management practices in pursuit of optimal patient care and outcomes.

**Table 2:** Nurses perception about the type of leadership across different hospitals and patient’s satisfaction.

	Hospitals				(N, %) *P*-value
	PMAH (n = 53) (A)	NGH (n = 4) (B)	KFMC (n = 48) (C)	PSMMC (n = 45) (D)	
Type of leadership	(N, %)	(N, %)	(N, %)	(N, %)	
Transformational	0	0	0	5 (11.1%)	5 (3.3) ND
Transactional	10 (18.8%)	3 (75%)	21 (43.7%)^ABD^	9 (20%)	43 (28.6) .001
Passive-avoidant	43 (81.1%)^BCD^	1 (25%)	27 (56.2%)	23 (51.1%)	94 (62.6) .001
Outcome leadership	0	0	0	8 (17.7%)	8 (5.3) ND
Patient satisfaction, *m* (SD)	1.7 (0.1)	3.1 (0.1)^ACD^	2.5 (0.3)^A^	2.4 (0.3)^A^	.001

Values are presented as N (%) for categorical variables and mean (standard deviation) for continuous variables; *statistically significant at P < .05*; superscripts (A, B, C, D) indicate statistically significant differences between groups based on post hoc analysis, where A denotes a significant difference from PMAH, B from NGH, C from KFMC, and D from PSMMC. Tests are adjusted for all pairwise comparisons within a row of each innermost sub-table using the Bonferroni correction.

KFMC = King Fahad Medical City, *m* (SD) = mean (standard deviation), N (%) = number, percentage, ND = not determined, NGH = National Guard Health Affairs, PMAH = Prince Mohammed Bin Abdulaziz Hospital, PSMMC = Prince Sultan Military Medical City.

In our study, we observed distinct dominant leadership traits across the 4 hospitals under investigation. Notably, at PMAH, the predominant leadership trait was identified as management by exception (passive) (MBRP) (*m* = 1.8, SD = 0.5), with statistically significant differences (*P* < .001) from the other hospitals. Conversely, at NGH, CR emerged as the prevailing leadership trait, (*m* = 1.9, SD = 0.4). KFMC showed a unique dominance of CR as the primary leadership trait, (*m* = 1.7, SD = 0.6). Lastly, PSMMC exhibited a distinctive combination of LF and management by exception (active) (MBEA) as the dominant leadership traits, (*m* = 1.5, SD = 0.6). These findings underscore the striking variations in leadership philosophies across these hospitals, with potential implications for organizational culture, staff motivation, and, ultimately, the quality of patient care (Table 3).

**Table 3:** Dominant leadership style in different hospitals.

Leadership style	Leadership traits	PMAH (n=53) (A)	NGH (n=4) (B)	KFMC (n=48) (C)	PSMMC (n=45) (D)
Transformational	IA	0.4 (0.4)	1.3 (0.7)^AC^	0.5 (0.3)	1.1 (0.8)^AC^
	IB	0.2 (0.3)	ND	0.1 (0.7)	1.1 (0.7)^ABC^
	IM	0.1 (0.2)	0.3 (0.3)	1.0 (0.6)^A^	1.0 (0.8)^A^
	IS	0.5 (0.3)	ND	0.1 (0.1)	1.0 (0.8)^ABC^
	IC	0.5 (0.4)	0.6 (0.1)	0.2 (0.2)	1.2 (0.7)^AC^
Transactional	CR	1.5 (0.3)	1.9 (0.4)	1.7 (0.6)^D^	1.4 (0.4)
	MBEA	0.8 (0.4)^C^	0.4 (0.5)	0.4 (0.4)	1.5 (0.6)^ABC^
Passive avoidant	MBRP	1.8 (0.5)^BCD^	1.1 (0.3)	1.4 (0.4)	1.3 (0.3)
	LF	1.1 (0.4)^C^	0.7 (0.6)	0.7 (0.4)	1.5 (0.6)^ABC^
Outcome leadership	EE	0.2 (0.2)	ND	0.1 (0.1)	0.8 (0.9)^ABC^
	EEF	0.6 (0.2)	0.5 (0.1)	0.5 (0.1)	1.2 (0.8)^AC^
	SAT	0.6 (0.5)	0.3 (0.2)	0.4 (0.3)	1.1 (0.0)^AC^

The superscripted alphabets represent the row comparison. Tests are adjusted for all pairwise comparisons within a row of each innermost subtable using the Bonferroni correction.

CR = contingent reward, EE = extra effort, EEF = effectiveness, IA = idealized attributes or idealized influence (attributes), IB = idealized attributes or idealized influence (behaviors), IC = individual consideration, IM = inspirational motivation, IS = intellectual stimulation, KFMC = King Fahad Medical City, LF = Laissez-Faire, MBEA = management by exception (active), MBRP = management by exception (passive), NGH = National Guard Hospital, PMAH = Prince Mohammad bin Abdulaziz Hospital, PSMMC = Prince Sultan Military Medical City, SAT = satisfaction.

The results of the ANOVA indicated a statistically significant difference in patient satisfaction scores among leadership styles (*F* [3, 176] = 25.421, *P* < .001). TS (*m* = 2.7, SD = 0.4) displayed the highest patient satisfaction scores, followed by outcome leadership (M = 2.5, SD = 0.23), TF (*m* = 2.2, SD = 0.19), and PA (*m* = 2.1, SD = 0.48). The post hoc multiple comparisons, adjusted for unequal variances using the Games-Howell procedure, revealed significant differences. Specifically, the TS exhibited significantly higher mean scores compared to the PA (MD = 0.60, SE = 0.071, *P* < .001, 95% CI = 0.42–0.79) and the TF (MD = 0.54, SE = 0.10, *P* = .004, 95% CI = 0.21–0.88). In contrast, the PA group had significantly lower mean scores than both the TS (MD = 0.60, SE = 0.07, *P* < .001, 95% CI = 0.42–0.79) and the OL (MD = 0.37, SE = 0.09, *P* = .008, 95% CI = 0.09–0.65). A sensitivity analysis was performed to investigate the association between leadership styles and patient satisfaction scores, incorporating demographic patient data as a covariate. The analysis revealed that the relationship between nurses’ perceived leadership style and patient satisfaction scores remained unchanged, as no significant alterations were observed (File 1, Supplemental Digital Content, http://links.lww.com/MD/O520).

## 4. Discussion

To the best of our knowledge, this study marks the first attempt to comprehensively identify the prevalent nursing leadership style and its impact on patient care quality in the Kingdom of Saudi Arabia. The research encompassed 4 well-regarded hospitals in the central region of Saudi Arabia. Among the nursing leadership styles observed in these 4 major hospitals, the most prevalent leadership style was the PA (n = 94, 62.6%), followed by the transactional style (n = 43, 28.6%), the outcome style (n = 8, 5.3%), and the least common was the transformational style (n = 5, 3.3%). Notably, the passive avoidance leadership style was associated with lower patient satisfaction, while the highest patient satisfaction was reported with transformational and TS styles. It is crucial to acknowledge the critical role of nurses within healthcare institutions and recognize that how they are led by their superiors can significantly affect their performance and, consequently, patient outcomes.

Multifactor leadership questionnaire staff form to gather insights from staff nurses regarding their managers’ leadership styles. The analysis indicates that the most prevalent leadership style across the 4 hospitals was the PA leadership style. Our research revealed that nurse managers predominantly employ a combination of transactional and passive avoidance leadership styles. Passive avoidance leadership is distinguished by the proclivity of a leader to refrain from assuming accountability, making determinations, or confronting matters. Frequently, it leads to disengagement, disregard for team development, and a deficiency in accountability. This particular approach has the potential to result in ineffective communication, unresolved conflicts, and diminished morale within the team. Passive avoidance leadership is widely regarded as ineffective and capable of yielding adverse outcomes for patient care^[16]^ and the overall work environment within the healthcare sector, including nursing.^[26]^ Similarly, a study reported the impact of different leadership styles of nurse leaders on job satisfaction in 3 private hospitals in Amman.^[27]^ The results show that TF had the highest positive influence on job satisfaction, followed by TS. In contrast, PA leadership had a negative impact on job satisfaction.^[27]^ This study reported Management by Exception (Passive) was the predominant PA leadership style. The utilization of Management by Exception (Passive) can prove to be successful in circumstances where nurses possess a high level of expertise, extensive experience, intrinsic motivation, and well-established organization’s operational procedures.^[28]^ Nevertheless, the situation can present difficulties when nurses necessitate additional direction, assistance, or motivation.^[29]^ A study conducted in 5 Italian hospitals involved 479 registered nurses and 829 patients.^[16]^ The study reported that nurse satisfaction with leadership was associated with reduced burnout, improved interpersonal relationships, decreased misbehavior, and ultimately, higher patient satisfaction with the quality of care.^[16]^ This underscores the importance of leadership and organizational context in influencing patients’ perceptions of care quality and suggests that healthcare managers should consider these factors to enhance patient care.^[16]^ A study involving a sample of 302 nursing home staff reported a predominance of PA leadership style and recommended the implementation of transformation or TS style.^[30]^ The findings of this study indicate that hospitals characterized by a predominant passive avoidance leadership style were associated with a low perceived level of patient satisfaction. The hospital with a combination of transformational and TS styles, resulted in a high level of patient satisfaction. Consistent with our findings, numerous research has reported a significant degree of patient contentment associated with both transformational and TS styles.^[31–34]^

Laissez-faire is a form of PA leadership style characterized by a lack of active leadership involvement and a tendency to avoid intervention. Leaders employing this style postpone decision-making, refrain from providing feedback, and do not acknowledge good performance. Our study identified the predominance of LF in PSMMC (*m* = 1.5, SD = 0.6) and PMAH (*m* = 1.1, SD = 0.4). It is noteworthy to mention that the PMAH had a low level of patient satisfaction whereas PSMMC showed moderate to high levels of patient satisfaction. This disparity could be due to leadership predominance in PSMMC being homogenously distributed across 4 identified leadership styles, the PMAH showed more inclination towards PA leadership style. In line with our results studies report that minimal effort is made in PA leadership style to motivate employees or recognize their contributions.^[19]^ LF leaders are notably absent when employees require guidance. Studies have demonstrated that this leadership approach correlates with reduced job satisfaction and lower contentment with one’s immediate supervisor.^[35]^

These findings align with previous research by Zaghini et al in 2020,^[16]^ supporting the notion that nurse satisfaction with leadership has a direct influence on patient care quality. Mendes and de Jesus José Gil Fradique study in 2014^[36]^ reported that nursing leadership has a significant impact on healthcare quality, similarly to Saleh et al in 2018,^[37]^ who reported leadership effects on nurse satisfaction, turnover, and patient care quality. The research by Sfantou et al in 2017^[38]^ reported a strong correlation between leadership styles and healthcare quality, emphasizing their critical role in enhancing quality measures in healthcare and nursing. The latter study additionally reported that leadership had a crucial role in shaping organizational culture and engaging patients and nurses.^[39]^ Further, a study also supported the idea that relationship-focused leadership behaviors improve patient outcomes and nursing care quality compared to task-focused behaviors.^[40]^

## 5. Limitations

Limitations of this study include its focus on a specific set of hospitals, potentially limiting the generalizability of the findings to a broader healthcare context. The reliance on self-reported data, such as staff nurses’ opinions about their managers’ leadership styles and patient satisfaction, may introduce response bias. Additionally, the study does not delve deeply into the contextual factors and organizational dynamics that may influence leadership styles or their effects, which could provide a more comprehensive understanding. Furthermore, the cross-sectional design of the study limits the ability to establish causality, and the reliance on numerical scores for leadership styles and satisfaction levels may oversimplify the complex nature of these constructs in the healthcare environment. Further research with a more diverse and larger sample, qualitative data, and longitudinal assessments is needed to address these limitations and offer a more comprehensive understanding of leadership’s impact on nurse and patient outcomes in healthcare settings (File 2, Supplemental Digital Content, http://links.lww.com/MD/O521 and File 3, Supplemental Digital Content, http://links.lww.com/MD/O522).

## 6. Recommendations for future studies

Future research in the field should focus on investigating leadership interventions that seek to transform the prevalent PA leadership styles observed among nurse managers in public hospitals in Saudi Arabia. Longitudinal studies that monitor the evolution of leadership styles and patient satisfaction over an extended time will yield valuable insights regarding long-lasting effects. Comparative analyses conducted within various healthcare settings have the potential to reveal the impact of cultural and contextual factors. Qualitative research methods, such as interviews and focus groups, have the potential to provide in-depth and nuanced insights. By conducting an examination of patient-centered care models and implementing a multi-stakeholder approach that includes physicians, administrative staff, and management, it is possible to enhance comprehension and provide practical suggestions for enhancing healthcare quality improvement.

## 7. Conclusion

In conclusion, our research highlights the multifaceted impact of leadership styles in healthcare. The results reinforce the pivotal role of nurse managers in the effectiveness of healthcare organizations and the quality of patient care. By adopting transformational and TS practices and improving service quality, healthcare institutions can further enhance both nurse and patient experiences, ultimately leading to improved healthcare outcomes.

## 8. Clinical implication

The prevalence of PA leadership styles among nurse managers is associated with the lowest levels of patient satisfaction. Conversely, a predominant TS style is associated with the highest patient satisfaction. This highlights the critical impact of nursing leadership styles on patient satisfaction and underscores the need for interventions and further research to develop strategies that can enhance leadership styles, ultimately leading to improved patient outcomes and healthcare quality.

## 9. Patient and nurse’s contribution

Patients serve as the primary data source, offering insights into their satisfaction through responses to the PSNCQQ, forming the basis for the assessment of patient satisfaction. On the other hand, nurses play a pivotal role by collecting data related to nursing leadership styles using the MLQ, thus providing critical information for analyzing the impact of leadership styles on patient satisfaction.

## Author contributions

**Conceptualization:** Amira Yahia Boshra, Fatima Aseeri, Sharifa Alasiry, Mehrunnisha Ahmad, Gopal Nambi, Mohammad Shaphe, Mohammad Sidiq, Faizan Kashoo.

**Data curation:** Amira Yahia Boshra, Fatima Aseeri, Sharifa Alasiry, Mehrunnisha Ahmad, Gopal Nambi, Mohammad Shaphe, Mohammad Sidiq, Abhishek Sharma, Faizan Kashoo.

**Formal analysis:** Amira Yahia Boshra, Mehrunnisha Ahmad, Aksh Chahal, Gopal Nambi, Abhishek Sharma, Faizan Kashoo.

**Funding acquisition:** Aksh Chahal.

**Investigation:** Sharifa Alasiry, Mehrunnisha Ahmad, Aksh chahal, Gopal Nambi, Mohammad Sidiq, Faizan Kashoo.

**Methodology:** Amira Yahia Boshra, Fatima Aseeri, Sharifa Alasiry, Mehrunnisha Ahmad, Aksh chahal, Mohammad Shaphe, Mohammad Sidiq, Abhishek Sharma, Faizan Kashoo.

**Project administration:** Mohammad Shaphe, Faizan Kashoo.

**Resources:** Fatima Aseeri, Mehrunnisha Ahmad, Mohammad Shaphe, Faizan Kashoo.

**Software:** Mehrunnisha Ahmad, Aksh chahal, Gopal Nambi, Mohammad Sidiq, Abhishek Sharma, Faizan Kashoo.

**Supervision:** Amira Yahia Boshra, Sharifa Alasiry, Mehrunnisha Ahmad, Gopal Nambi, Mohammad Shaphe, Abhishek Sharma, Faizan Kashoo.

**Validation:** Fatima Aseeri, Gopal Nambi.

**Visualization:** Fatima Aseeri.

**Writing – original draft:** Amira Yahia Boshra, Sharifa Alasiry, Mehrunnisha Ahmad, Aksh chahal, Gopal Nambi, Mohammad Shaphe, Mohammad Sidiq, Abhishek Sharma, Faizan Kashoo.

**Writing – review & editing:** Amira Yahia Boshra, Fatima Aseeri, Sharifa Alasiry, Aksh Chahal, Gopal Nambi, Mohammad Shaphe, Mohammad Sidiq, Abhishek Sharma, faizan kashoo.

## Supplementary Material

SUPPLEMENTARY MATERIAL
