# Supplementary material for: Impact of leadership styles on patient satisfaction with nursing care quality in public hospitals: A cross-sectional study
Source: Medicine (Baltimore). 2025 Mar 14;104(11):e41670. doi: 10.1097/MD.0000000000041670 (PMC11922418; doi:10.1097/MD.0000000000041670)
Supplement: SUPPLEMENTARY MATERIAL [file medi-104-e41670-s001.pdf]

Univariate Analysis of Variance

[DataSet1] C:\Users\sady3\OneDrive\Desktop\Ameera research on leadership\Submission Article\Faizan Revision\Analysis for quality of care from patients.sav

Between-Subjects Factors

		Value Label	N
Leadership	1	TS	77
	2	PA	90
	3	TF	5
	4	OL	8

Descriptive Statistics

Dependent Variable: Scoremean

Leadership	Mean	Std. Deviation	N
TS	2.7567	.43686	77
PA	2.1497	.48546	90
TF	2.2105	.19693	5
OL	2.5263	.23028	8
Total	2.4278	.53694	180

Levene's Test of Equality of Error Variances[a]

Dependent Variable: Scoremean

F	df1	df2	Sig.
.186	3	176	.906

Tests the null hypothesis that the error variance of the dependent variable is equal across groups.

a. Design: Intercept + Gender + Maritalstatus + Hospital + Age + Healthbefore + overallperception + Qualityofnursingcare + Healthafter + recommededto + Leadership

Tests of Between-Subjects Effects

Dependent Variable: Scoremean

Source	Type III Sum of Squares	df	Mean Square	F	Sig.
Corrected Model	35.826[a]	12	2.985	31.593	.000
Intercept	1.096	1	1.096	11.598	.001
Gender	.019	1	.019	.204	.652
Maritalstatus	.115	1	.115	1.221	.271
Hospital	3.656	1	3.656	38.693	.000
Age	.084	1	.084	.894	.346
Healthbefore	.172	1	.172	1.821	.179
overallperception	.657	1	.657	6.957	.009
Qualityofnursingcare	.477	1	.477	5.050	.026
Healthafter	1.762	1	1.762	18.645	.000
recommededto	1.458	1	1.458	15.431	.000
Leadership	2.854	3	.951	10.066	.000
Error	15.781	167	.094		
Total	1112.546	180			
Corrected Total	51.607	179			

Tests of Between-Subjects Effects

Dependent Variable: Scoremean

Source	Partial Eta Squared	Noncent. Parameter	Observed Power[b]
Corrected Model	.694	379.114	1.000
Intercept	.065	11.598	.923
Gender	.001	.204	.073
Maritalstatus	.007	1.221	.196
Hospital	.188	38.693	1.000
Age	.005	.894	.156
Healthbefore	.011	1.821	.269
overallperception	.040	6.957	.746
Qualityofnursingcare	.029	5.050	.608
Healthafter	.100	18.645	.990
recommededto	.085	15.431	.974
Leadership	.153	30.197	.998
Error			
Total			
Corrected Total			

a. R Squared = .694 (Adjusted R Squared = .672)

b. Computed using alpha = .05

Estimated Marginal Means

1. Grand Mean

Dependent Variable: Scoremean

Mean	Std. Error	95% Confidence Interval	
		Lower Bound	Upper Bound
2.310[a]	.051	2.210	2.410

a. Covariates appearing in the model are evaluated at the following values: Gender = 1.5833, Maritalstatus = 1.5278, Hospital = 2.4944, Age = 34.9167, Healthbefore = 3.0722, overallperception = 2.5500, Qualityofnursingcare = 2.6278, Healthafter = 2.4778, Recommended to friend and family = 2.0889.

2. Leadership

Estimates

Dependent Variable: Scoremean

Leadership	Mean	Std. Error	95% Confidence Interval	
			Lower Bound	Upper Bound
TS	2.589[a]	.038	2.514	2.665
PA	2.324[a]	.035	2.255	2.394
TF	2.064[a]	.147	1.775	2.354
OL	2.262[a]	.122	2.022	2.502

a. Covariates appearing in the model are evaluated at the following values: Gender = 1.5833, Maritalstatus = 1.5278, Hospital = 2.4944, Age = 34.9167, Healthbefore = 3.0722, overallperception = 2.5500, Qualityofnursingcare = 2.6278, Healthafter = 2.4778, Recommended to friend and family = 2.0889.

Pairwise Comparisons

Dependent Variable: Scoremean

(I) Leadership	(J) Leadership	Mean Difference (I-J)	Std. Error	Sig.[b]	95% Confidence [b]...
					Lower Bound
TS	PA	.265*	.055	.000	.117
	TF	.525*	.153	.005	.116
	OL	.328	.129	.072	-.017
PA	TS	-.265*	.055	.000	-.413
	TF	.260	.151	.526	-.144
	OL	.063	.128	1.000	-.280
TF	TS	-.525*	.153	.005	-.935
	PA	-.260	.151	.526	-.665
	OL	-.198	.179	1.000	-.675
OL	TS	-.328	.129	.072	-.672
	PA	-.063	.128	1.000	-.405
	TF	.198	.179	1.000	-.280

Pairwise Comparisons

Dependent Variable: Scoremean

(I) Leadership	(J) Leadership	95% Confidence [b]...
		Upper Bound
TS	PA	.413
	TF	.935
	OL	.672
PA	TS	-.117
	TF	.665
	OL	.405
TF	TS	-.116
	PA	.144
	OL	.280
OL	TS	.017
	PA	.280
	TF	.675

Based on estimated marginal means

*. The mean difference is significant at the .05 level.

b. Adjustment for multiple comparisons: Bonferroni.

Univariate Tests

Dependent Variable: Scoremean

	Sum of Squares	df	Mean Square	F	Sig.	Partial Eta Squared
Contrast	2.854	3	.951	10.066	.000	.153
Error	15.781	167	.094			

Univariate Tests

Dependent Variable: Scoremean

	Noncent. Parameter	Observed Power[a]
Contrast	30.197	.998
Error		

The F tests the effect of Leadership. This test is based on the linearly independent pairwise comparisons among the estimated marginal means.

a. Computed using alpha = .05

Spread-versus-Level Plots

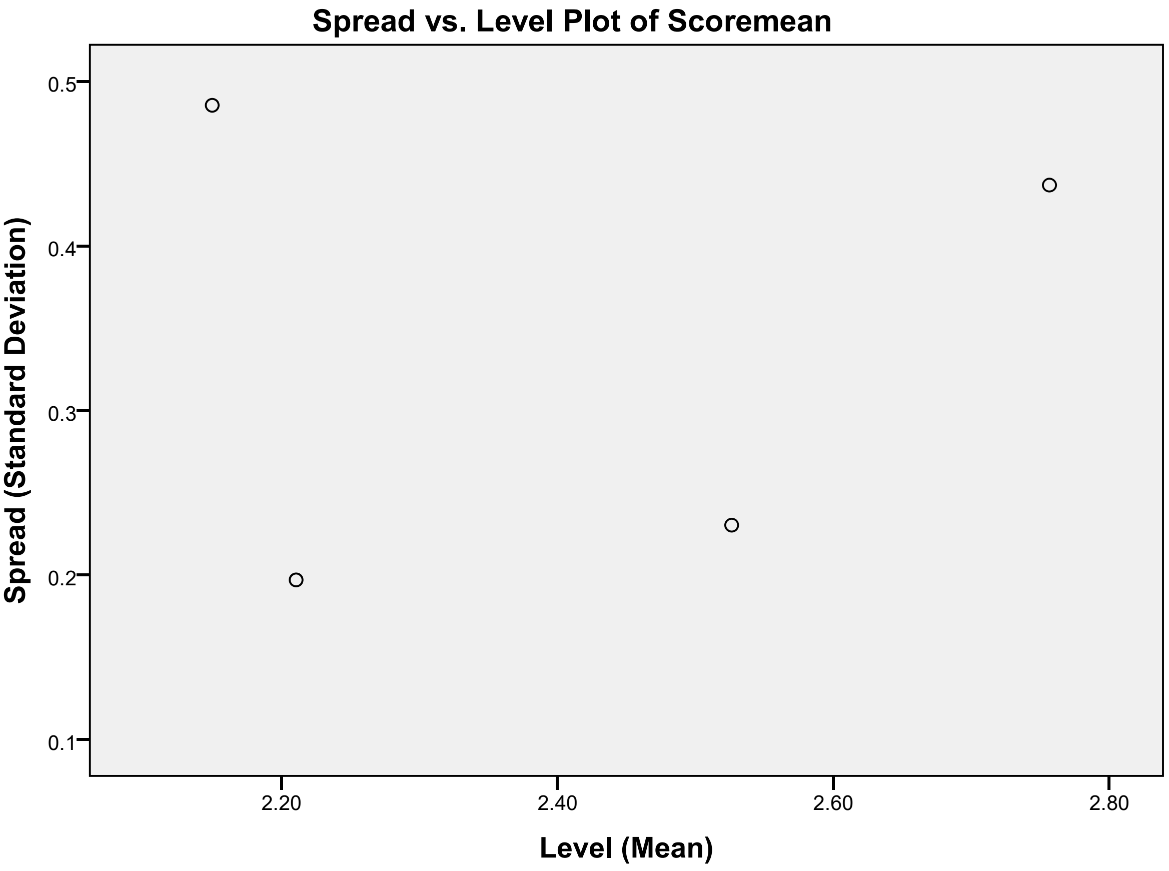

Groups: Leadership

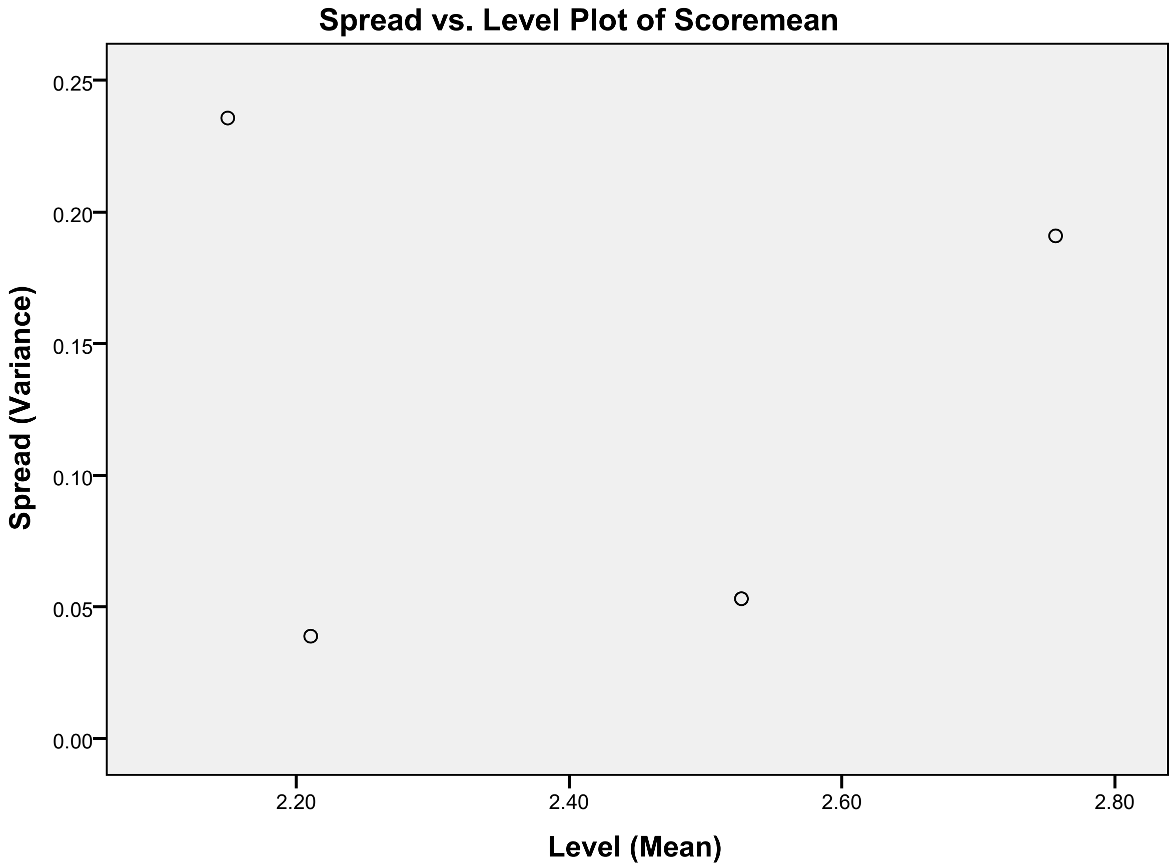

Groups: Leadership
